# The Role of Fatty Acids in Non-Alcoholic Fatty Liver Disease Progression: An Update

**DOI:** 10.3390/ijms22136900

**Published:** 2021-06-27

**Authors:** Aleksandra Hliwa, Bruno Ramos-Molina, Dariusz Laski, Adriana Mika, Tomasz Sledzinski

**Affiliations:** 1 Department of Pharmaceutical Biochemistry, Faculty of Pharmacy, Medical University of Gdansk, Debinki 1, 80-211 Gdansk, Poland; aleksandra.hliwa@gumed.edu.pl (A.H.); adriana.mika@gumed.edu.pl (A.M.); 2 Obesity and Metabolism Group, Biomedical Research Institute of Murcia (IMIB-Arrixaca), 30120 Murcia, Spain; brunoramosmolina@gmail.com; 3 Department of General, Endocrine and Transplant Surgery, Faculty of Medicine, Medical University of Gdansk, Smoluchowskiego 17, 80-214 Gdansk, Poland; dariusz.laski@gumed.edu.pl

**Keywords:** non-alcoholic fatty liver disease, lipids, lipidomics, fatty acids

## Abstract

Non-alcoholic fatty liver disease (NAFLD) is a major public health problem worldwide. NAFLD (both simple steatosis and steatohepatitis) is characterized by alterations in hepatic lipid metabolism, which may lead to the development of severe liver complications including cirrhosis and hepatocellular carcinoma. Thus, an exhaustive examination of lipid disorders in the liver of NAFLD patients is much needed. Mass spectrometry-based lipidomics platforms allow for in-depth analysis of lipid alterations in a number of human diseases, including NAFLD. This review summarizes the current research on lipid alterations associated with NAFLD and related complications, with special emphasis on the changes in long-chain and short-chain fatty acids levels in both serum and liver tissue, as well as in the hepatic expression of genes encoding the enzymes catalyzing lipid interconversions.

## 1. Introduction

In the last decades, chronic liver disorders have become a major public health problem worldwide. Obesity and metabolic syndrome are well-known risk factors in chronic liver diseases (CLD). Over 400 million people struggle with obesity, and it is estimated that 75% of them will develop non-alcoholic fatty liver disease (NAFLD) [1]. NAFLD encompasses a spectrum of liver infractions, ranging from excessive storage of fat in the organ (simple steatosis) to non-alcoholic steatohepatitis (NASH). NASH differs from simple steatosis (SS) by the presence of hepatocyte death and inflammation and it is highly related to the development of advanced liver diseases such as fibrosis, cirrhosis, and even hepatocellular carcinoma (HCC) [1,2]. NAFLD can be associated with insulin resistance, which causes exaggerated lipolysis in the adipose tissue. As a result, there is an increase in the levels of serum free fatty acids (FFA) and hepatic triacylglycerols (TAG) [3]. It could also arise from congenital disorders and/or quick body mass loss after bariatric surgery [4,5,6]. Moreover, fatty liver is a common adverse effect of some medications (i.e., tamoxifen) [1,3].

The global prevalence of NAFLD has been estimated to be 25%, and it is not equally distributed in all parts of the world. In fact, more cases are reported in Western countries and the United States [7]. However, current data show the highest prevalence of NAFLD in South America [8]. Nevertheless, because of the lack of specific and non-invasive tests for the assessment of the severity of NAFLD, these data should be interpreted with caution. In clinical practice, NASH is mostly diagnosed based on the histopathological analysis of liver biopsies, which is an invasive procedure. Due to the procedure’s risks, a liver biopsy is usually performed in the case of significant symptoms suggesting NASH; therefore it cannot have a significant role in either prevention or early detection of NASH. Therefore, new non-invasive methods should be developed for the discrimination of patients with SS from those with NASH, as the latter represents an important risk factor for more severe liver complications such as cirrhosis and HCC. The accumulation of lipids leads to large-droplet macrovesicular steatosis and irreversible small-droplet steatosis [9]. Another liver injury associated to NAFLD is hepatocyte ballooning, which is characterized by swollen hepatocytes with a rarified cytoplasm [7]. Changes in the micro-architecture of tissue structure lead to increased resistance to blood flow through the liver and portal hypertension. The last step of cirrhosis is progressive, constant, and unstoppable fibrosis, which makes liver functions inefficient [9]. Liver cirrhosis is an ideal condition predisposing to the development of HCC, and it is estimated that nearly 80% of cirrhotic patients will develop HCC [9].

Lipids are a very heterogeneous group of organic compounds with different functions, e.g., energy storage and the formation and stabilization of intra- and extracellular membranes. Lipids are also precursors of bioactive molecules present in the circulation and they can even regulate genes expression [10,11]. Fatty acids (FA) are components of nearly every existing lipid structure. In the human body, FAs can be present in standalone form (FFAs) and in esterified form, e.g., in TAG and phospholipids (PL). Notably, FFA could be a misleading term, as these molecules are usually bound to albumin or other FA-binding proteins in the circulation.

**Figure 1:**
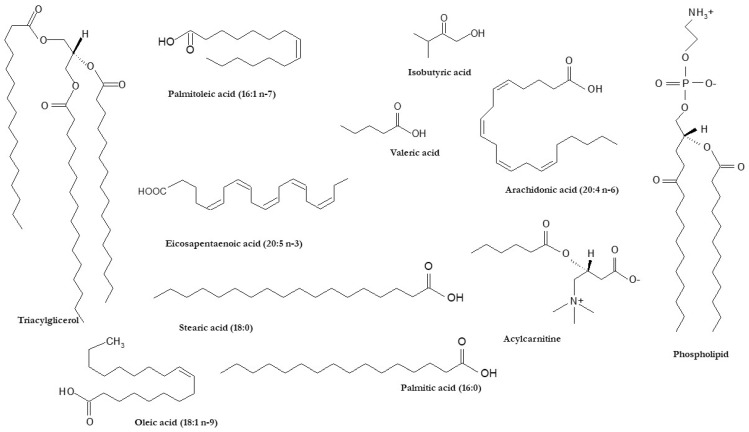
Chemical structures of FAs and lipid species including FAs in their backbone associated with human chronic liver disease.

Whereas a significant proportion of FA species can be synthesized by the human body, FAs are also taken up from the diet or even produced by the gut microbiota (i.e., short-chain FAs) [12]. The major FA synthetized de novo in human tissues (mainly in the liver) is palmitic acid (16:0), a saturated FA (SFA). By contrast, FA species with at least one double bond inserted in the acyl chain are defined as unsaturated FAs. The enzymes responsible for this process are called desaturases and include stearoyl-CoA desaturase (SCD1), which is present in the liver and synthesizes oleic acid (18:1 n-9) from stearic acid (18:0); oleic acid is the main component of TAGs [13]. Depending on the number of double bonds, unsaturated FAs can be classified as monounsaturated (MUFAs) and polyunsaturated FAs (PUFAs). PUFAs, which cannot be synthesized de novo in the human body, exert different bioactivities depending on the location of the double bonds. For instance, n-3 PUFAs can lead to various biological effects, as they are able to change the composition of plasma membranes as well as to modulate gene expression and certain cell signaling pathways [13,14]. N-3 PUFAs also play a significant protective role for chronic diseases, including cardiovascular disease (CVD), type 2 diabetes, and even cancers [13,14]. N-3 PUFAs are precursors of various lipid mediators with anti-inflammatory potential such as eicosanoids, resolvins and protectins [13,14]. In addition, n-3 PUFAs are considered key factors in the prevention of some undesirable bodily reactions, such as autoimmune response [13,14]. In contrast, pro-inflammatory oxylipins, including eicosanoids, are mostly produced from n-6 PUFAs [13,14]. The structures of FAs and complex lipids including FA in their structure with reported alterations in human chronic liver diseases (i.e., NAFLD) are presented in Figure 1.

Studies based on lipidomics approaches provide essential data on the changes of a wide range of lipid species [15] and can significantly contribute to extending the knowledge of lipid alterations associated with NAFLD pathogenesis. In this review, we specifically focused on the profile of FAs, the main components of almost all lipid species, in NAFLD pathogenesis. In the following paragraphs, we summarize the current evidence on: (1) the changes in the FA profile in both liver tissue and serum of NAFLD patients; (2) the alterations in the expression levels of genes involved in hepatic lipid metabolism in the onset of NAFLD; (3) the role of gut microbiota-derived short-chain FAs in NAFLD; (4) the lipid disorders linked to HCC in NASH patients.

## 2. Alterations of Hepatic FA Profile in NAFLD Pathogenesis

The lipidomic landscape of NAFLD is not well defined. The analysis of the alterations in hepatic and plasma lipid homeostasis in NAFLD patients could provide essential evidence on the pathophysiological hallmark of NAFLD [16,17]. Several lipidomic studies of liver tissue samples from NAFLD patients revealed a significant increase in hepatic TAG levels [18,19,20,21]. In addition to TAG, diacylglycerols (DAG), which are highly related to hepatic insulin resistance [22,23], have been also reported in higher concentrations in NAFLD livers [21,24]. On the other hand, it is believed that elevated circulating FFAs could be a major cause of hepatic lipotoxicity and concomitant hepatocytes injury [25]. Nevertheless, as stated above, not every FA fraction is harmless. Thus, liver damage has been specifically ascribed to toxic effects of SFA (especially, 16:0) accumulation in this organ. The ratios of specific FAs are dependent not only on diet and lipogenesis de novo, but also on sex and age. For instance, Yamada et al. reported that men exhibited more significant changes when comparing SS and NASH patients, than postmenopausal women [26]. Increased levels of hepatic SFAs were reported in both sexes of NAFLD patients [16,27]. In in vitro studies, SFAs have been shown to induce the synthesis of proinflammatory cytokines, leading to apoptosis and impaired insulin signal patches [27,28]. Excessive SFA (especially, 16:0 and 18:0) accumulation in hepatocytes is able to induce endoplasmic reticulum stress [23,29] and can represent a major cause of hepatocyte injury [27]. Furthermore, animal models of NAFLD displayed higher hepatic SFA concentrations compared to the control group, this difference being largely due to significant increases in myristic (14:0), palmitic (16:0), and stearic (18:0) acids [17]. A similar pattern of increased SFAs was observed in a study conducted in NASH patients [30]. Conversely, another lipidomic study in human livers showed a non-significant trend of higher SFAs, with only a significant increase in 16:0 among the different FA species included in the analysis [21]. Notably, a high content of 16:0 and an altered balance between 18:0 and 16:0 have been associated with hepatocyte ballooning in patients suffering from NASH [26].

In addition to SFAs, MUFAs were also elevated in liver tissue samples in the course of NAFLD [17,21,26,30]. An enhanced MUFAs mole percentage in DAG, TAG, and phospholipids has been reported in NAFLD livers [17,19,21]. Significantly higher concentrations of palmitoleic acid (16:1 n7) and oleic acid (18:1 n9) were found in human [21,30] and mouse NAFLD liver tissue [17]. Interestingly, elevated ratios of 16:1 n7/16:0 and 18:1 n9/18:0 were reported in NAFLD patients [21,26,30], which suggests an increase of SCD1 activity in NAFLD conditions [17,21,26].

Additionally, lipidomic analyses revealed a progressive decrease of hepatic PUFAs in parallel to the severity of NAFLD [21,30]. Thus, the molar percentages of both n-3 and n-6 PUFAs were decreased in human liver biopsies from NAFLD patients [21], while only n-3 PUFA content was lowered significantly in the TAG fraction. Accordingly, the n-6/n-3 ratio was increased in the liver tissue of patients with hepatic steatosis and NASH, whereas it was decreased in livers of mice with experimental NAFLD [17,21,26]. These discrepancies between humans and studies in experimental models with NAFLD suggest that the latter may not be a good model to study PUFA metabolism in human NAFLD. Moreover, the levels of eicosapentaenoic acid (20:5 n-3) and docosahexaenoic acid (22:6 n-3) were reported to be significantly decreased in NASH [21,30]. In addition, there was a significant depletion of gamma-linolenic acid (18:3 n-6) and arachidonic acid (20:4 n-6) [21], which could suggest their excessive utilization to synthesize inflammatory factors that would contribute to NAFLD pathogenesis [21,30]. Importantly, n-3 PUFAs are the precursors of cell PL, which could partially explain the lower PL amounts observed in NASH patients [30].

Diet is another key factor involved in the modulation of the hepatic FAs. Epidemiological studies have shown that patients with NASH claim a diet richer in fat and poorer in complex carbohydrates and protein that the general population [31]. Several animal studies suggested that canola and krill oils, that are rich in n-3 PUFAs, may prevent the onset of NAFLD, although it is still unclear if diets rich in n-3 PUFAs may reverse NAFLD in humans [31]. In this regard, other authors suggested that unsaturated fat intake has some beneficial effects, reserving that the use of dietary supplements including n-3 FAs needs further research before their recommendation for NAFLD patients [32].

## 3. Circulating FAs as Potential Biomarkers of NAFLD

As it was mentioned before, the number of non-invasive diagnostic tests for NAFLD is very limited. Screening for markers of liver injury is becoming a standard protocol for patients suffering from metabolic disorders including obesity, insulin resistance, or type 2 diabetes. Thus, the levels of serum transaminases (alanine aminotransferase and aspartate transaminase) only indicate damage in hepatocytes, but they are not sensitive enough to serve as a reliable non-invasive test in NAFLD patients [33,34,35]. Tests determining the levels of adipokines (i.e., adiponectin), hepatokines (i.e., FGF21), or certain pro-inflammatory cytokines have been shown to have better sensitivity to predict the status of NAFLD, albeit the available data are still limited and need further validation before their implementation in clinical laboratories [36,37]. Another potential biomarker is CK-18, whose fragments are released into the circulation as a result of hepatocyte death; the determination of CK-18 has shown promising results for the discrimination of the SS from NASH, but again, it needs further validation in larger patient cohorts [36].

An early and accurate detection of liver steatosis is of great interest because of the association between NAFLD and cardiovascular events and other metabolic diseases. Today, the quantification of liver steatosis can be performed by several ultrasound techniques. Controlled attenuation parameter (CAP) is the technique available in the FibroScan system (Echosens, Paris, France) that measures the attenuation of the ultrasound beam as it traverses the liver tissue [38]. CAP is evaluated together with liver stiffness measurements. This technique is promising but still being evaluated, and more research is needed. Despite the utility of ultrasound examination and CAP to diagnose hepatic steatosis, these techniques are not able to discriminate hepatocyte damage from inflammation, so they cannot be used for NASH diagnosis. So far, the most efficient medical imaging technique to diagnose the severity of NAFLD is computed tomography or magnetic resonance, but they are an expensive procedures and, in the case of computer tomography, they expose patient to X-rays [20], so they cannot be recommended for population screening programs.

Emerging evidence has proposed the potential use of metabolomic approaches to search for new non-invasive biomarkers of NAFLD [39]. In particular, plasma FAs levels in NAFLD subjects have been assessed in several lipidomic studies [40,41,42,43]. Overall, there is not a linear correlation for the levels of FAs between liver and blood, but the serum content can reflect metabolic changes in hepatic cells [41,43]. In 2017, Feng et al. proposed a panel of serum FFAs for the early diagnosis of NAFLD [42]. Interestingly, NAFLD patients had higher serum concentrations of all examined types of FFA, and there were no significant differences between total FFAs in lean or overweight patients with NAFLD [42]. In addition, this study revealed that obese NAFLD patients exhibited not only the highest amounts of serum FFAs, but also significantly higher 14:0, 16:0, 16:1, and 18:1 lipids with respect to lean subjects with NAFLD and healthy controls [42]. Moreover, even in lean NAFLD subjects, the abovementioned FA were significantly elevated [42]. Among all changes in serum FFA profiles observed in patients with NAFLD, researchers highlighted the potential use of 14:0 and 16:1 lipids as possible biomarkers of early NAFLD diagnosis. FA 14:0, which can originate from the diet or be synthetized endogenously, can induce proinflammatory reactions and consequently increase the risk of cardiovascular diseases [44]. The complex lipids (which all, except steroids non-esterified with FAs, contain FA in their structure) also have diagnostic potential. Ismail et al. [45] performed untargeted lipidomic analysis in blood samples, detecting more than 500 significantly different lipid species when patients with CLD were compared to healthy controls. Notably, TAG, PCs, and plasmalogens were the most significantly upregulated lipid species in the blood of CLD patients with respect to the controls.

## 4. Alterations of the Expression of Genes Related to NAFLD

The molecular mechanisms of NAFLD progression are still not completely understood. It is assumed that pathological changes are strictly related to chronic inflammation, insulin resistance, increased intrahepatic TAG accumulation, and de novo lipogenesis (DNL). In NAFLD patients, similar to healthy subjects, lipogenesis increases dramatically in the postprandial state [46]. The contribution of hepatic DNL to the content of palmitate in serum was estimated to be more than twice higher in patients with NASH than in healthy subjects [47]. DNL is a multi-step process carried out in the cytosol by specific enzymes. It is initiated by the first isoform of acetyl-CoA carboxylase (ACC1), which transforms acetyl-CoA into malonyl-CoA. At the mRNA level, ACC1 was almost two times higher in liver biopsies of NAFLD patients than in samples from subjects with normal liver function [48]. In other tissues (i.e., muscle), a second ACC isoform (ACC2) produces malonyl-CoA that inhibits carnitine palmitoyltransferase 1 (CPT1) and lowers FA oxidation. Therefore, the inhibition of both isoforms has been suggested as a potential therapeutic strategy to decrease systemic FA amounts [49,50,51]. Unexpectedly, the administration of specific ACC inhibitors to both rodents and humans resulted in a higher content of TAG in blood [51,52]. The mRNA levels of fatty acid synthase (FASN), another lipogenic enzyme, were also increased in the livers of obese and NASH patients [26,48], which could explain the excessive amounts of its product—palmitate. The crucial enzyme implicated in the regulation of lipid metabolism in the liver is SCD1, as its product oleoyl-CoA is a major substrate for TAG synthesis [53]. The mammalian SCD1 is involved in the pathogenesis of many disorders interrelated with metabolic syndrome [54]. Animal studies revealed that SCD1 deficiency leads to improvement of insulin sensitivity, decreased lipogenesis, and increases FA oxidation in the liver, which protect the animals from diet-induced obesity and hepatic insulin resistance [55,56,57] As mentioned above, NAFLD patients display elevated ratios of 16:1 n7/16:0 and 18:1 n9/18:0 [21,26,30], which suggests increased liver SCD1 activity. Hepatic SCD1 mRNA levels were significantly higher in NASH vs. SS patients. Moreover, a statistically significant correlation was observed between the lobular inflammation score and hepatic SCD1 mRNA expression [26]. CPT1 expression in liver biopsies of NAFLD patients was also decreased, which may suggest altered FA oxidation [37].

The sterol regulatory element-binding proteins (SREBPs) are a family of transcription factors (SREBP-1a, SREBP-1c, and SREBP-2) that activate the synthesis of FAs, TAGs, and cholesterol [31,58]. Exaggerated activation of SREBPs provokes TAG hepatic accumulation, which leads to steatosis [31] and further liver damage. In the liver, de novo FAs synthesis is induced by SREBP-1c. On the contrary, FA overabundance results in the downregulation of SREBP-1c in normal hepatocytes [59]. SREBP-1c gene expression was two times higher in NAFLD livers than in normal tissue, similar to its downstream positively regulated lipogenic enzyme genes—*ACC* and *FASN* [48]. Remarkably, *SREBP-1c* gene expression was significantly correlated with ballooning and fibrosis score in NASH patients [26].

The mechanisms by which alterations in the hepatic mRNA levels of genes involved in lipid metabolism may occur are not well understood. Interestingly, circulating FAs can act in a hormone-like manner on hepatocytes, and some of them (e.g., oleic acid or cyclopropaneoctanoic acid 2-hexyl) can induce hepatic lipogenesis [60,61]. Recent studies indicated that some regulators of FA metabolism, such as peroxisome proliferator-activated receptor α (PPAR-α) and sirtuin-1 (SIRT1), are targets of several miRNAs [62,63], suggesting that certain epigenetic mechanisms may also contribute to NAFLD-related hepatic lipid alterations.

**Figure 2:**
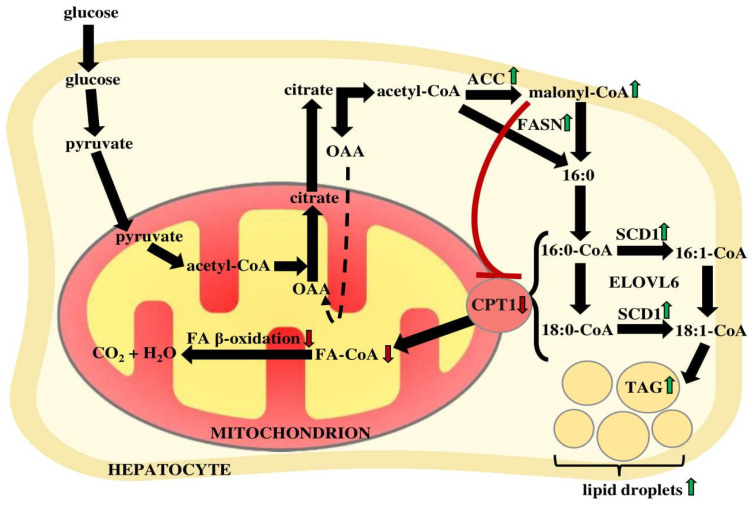
Lipid alterations in hepatocytes of patients with NAFLD. Lipid accumulation is a result of: (1) increased expression of the enzymes of lipid synthesis (acetyl-CoA carboxylase—ACC and fatty acid synthase—FASN) and desaturation (stearoyl-CoA desaturase—SCD1); (2) inhibition of carnitine palmitoyltransferase 1 (CPT1), which limits the transport of FA from the cytosol to the mitochondria, when they undergo beta-oxidation by excessive level of malonyl-CoA. FA—fatty acid, OAA—oxaloacetate, TAG—triacylglycerol.

The above discussed NAFLD-related lipid alterations and their molecular mechanism based on the changes of the expression of genes encoding enzymes of lipid metabolism are summarized in Figure 2.

Patatin-like phospholipase domain-containing 3 (*PNPLA3*) gene polymorphisms are associated with NASH, NAFLD, and even NAFLD-related HCC [64,65,66]. In obese subjects with NAFLD, hepatic PNPLA3 mRNA expression strongly correlated with hepatic TAG and DAG accumulation [64]. The most studied variant of the *PNPLA3* gene is I148M and it has been associated with an increase in hepatocellular lipid retention by altering TAG hydrolysis [67]. Due to that, intrahepatic TAGs are increased and cannot be metabolized. The I148M variant has been also associated with lower levels of circulating adiponectin, which exhibits anti-inflammatory and anti-fibrotic properties [67]. Considering those results, it can be assumed that the *PNPLA3* gene is involved in NAFLD development and progression.

## 5. Gut Microbiota-Derived Short-Chain Fatty Acids and NAFLD

The microbiota is composed of different bacterial populations with a mutualistic relationship that reside in the epithelial barriers of different organs in the host. Microbiota is a metabolically active ecosystem that interacts with epithelial and stromal cells, with a critical role in human health. Thus, when a balanced interaction between the gastrointestinal tract and the resident microbiota is disrupted, intestinal and extraintestinal diseases may develop. This includes metabolic disorders such as diabetes, cardiovascular dyslipidemia, or NAFLD. Emerging evidence suggests that alterations in the gut microbiota (dysbiosis) may play a role in NAFLD development and progression [68,69,70]. Diverse studies have shown that the gut microbiota can contribute to NAFLD pathogenesis through various mechanisms including the regulation of energy homeostasis and lipid metabolism in the liver, the modulation of bile acid metabolism and signaling, the endogenous ethanol production and LPS-mediated induction of pro-inflammatory cytokines by liver macrophages.

Short-chain fatty acids (SCFAs) are the main products of bacterial fermentation of dietary fibers, along with proteins and peptides that have escaped digestion by host enzymes in the upper gut. A growing body of work has identified SCFAs as mediators of diet-induced crosstalk between the microbiome and the host and that these microbial metabolites are important for health [71]. The most abundant SCFAs present in the colon lumen are acetate (2:0), propionate (3:0), and butyrate (4:0). SCFAs not only provide energy for the intestinal epithelium, but also have many bioactive roles, such as the regulation of immunity, lipometabolism, and glycometabolism, and the maintenance of gut microbiota homeostasis. Bacterial-derived SCFAs have been involved in liver function after their absorption and delivery to the liver via the portal vein. SCFAs could regulate hepatic lipid metabolism, and emerging evidence has suggested that they exert beneficial effects on metabolic liver diseases including NAFLD. Remarkably, dietary SCFAs ameliorate hepatic steatosis and insulin resistance in diet-induced obese mice, through a reduction in intrahepatic lipid accumulation [72,73,74]. SCFA-fed mice displayed reduced hepatic and protein expression of lipogenic enzymes [74,75,76,77]. Gut-derived SCFAs have also been reported to have a direct impact on FA synthesis, with only mild regulatory effects on the expression of genes involved in hepatic lipid metabolism [78]. In addition to hepatic lipogenesis, dietary SCFAs can downregulate hepatic cholesterol synthesis, contributing to lower plasma cholesterol [79]. Supplementation of SCFAs also exerts anti-inflammatory effects and attenuates NASH by restoring dysbiosis of the gut microbiota [75,80].

## 6. Hepatocellular Carcinoma and Lipid Alterations

Primary liver cancer was the sixth most commonly diagnosed cancer and the third leading cause of cancer death worldwide in 2020, with approximately 906,000 new cases and 830,000 death [81]. The most common type of primary liver cancer is HCC, representing the third leading cause of cancer-related deaths worldwide [82]. Depending on the world region, the main risk factors of HCC are viral hepatitis (HCV, HBV), aflatoxin-B1 exposure [83], alcoholic-related liver disease (ARLD), and NAFLD [45,82,84]. Importantly, NAFLD is becoming the leading HCC cause in both obese patients and non-obese individuals with metabolic disorders [85]. In addition, NAFLD has become a major cause of HCC-related mortality [86]. Due to the worldwide epidemic of obesity and type 2 diabetes mellitus, there is an overgrowing population of patients predisposed to NAFLD-related liver cirrhosis, liver insufficiency, and HCC [87]. Patients with obesity and NAFLD are characterized by an excessive intake of dietary FAs and have increased lipolysis in visceral adipose tissue that is caused by insulin resistance. This results in elevated FA supply to hepatocytes. This condition is also characteristic for patients with HCC related to obesity and advanced NAFLD, albeit the mechanism by which this association occurs is not well understood [84].

As mentioned above, the liver is one of the main organs involved in the regulation of lipid metabolism. Therefore, alterations of liver function are directly associated with disturbances in lipid homeostasis. The biosynthesis of FA is enhanced in HCC and thus could potentially serve as a therapeutic target, together with additional lipid metabolism alterations that promote adaptation to the local environment in HCC [84]. Severe dysregulation of FA metabolism in HCC cells in comparison to normal/healthy hepatocytes is an effect of the association between oncogenic signaling pathways and the altered expression and activity of enzymes implicated in lipid metabolism [88]. Remarkably, several key enzymes of lipid metabolism have been related to cancer survival and may have a prognostic potential as biomarkers of cancer [89]. Metabolites produced during the metabolic reprogramming of hepatocytes into HCC cells (including FAs) can promote cancer formation through changes of signaling pathways, epigenetics, and cellular differentiation [84].

According to many authors, the association between blood and tumor lipidomes is still little known [45]. However, certain mass spectrometry-based lipidomics studies have provided key information about the potential metabolic alterations observed in HCC patients. For instance, an increase in FFA 16:1 [90] and a decrease in the FA composition (18:2 n-6, 20:4 n-6, 16:0 and 18:1 n-9) in the plasma phospholipids of HCC patients have been reported [91]. In turn, in a NASH-associated HCC mouse model, reduced levels of FFAs 18:3 n-3, 20:5 n-3, 22:6 n-3, and 18:2 n-6 were found [92]. An untargeted lipidomic analysis revealed that the blood levels of almost all lipid groups were decreased in HCC patients when compared to CLD subjects and that many specific lipid classes were up- or dowregulated in HCC patients with respect to healthy controls [45]. Other research showed significantly lower levels of PCs, PSs, and PIs in the serum of HCC patients [93]. Interestingly, in HCC tumor tissue, most lipid groups were decreased in comparison to non-tumor hepatic tissue [45], which is intriguing, because other studies suggest enhanced FA biosynthesis in HCC tumors [51]. However, it has been proposed that in some other cancers, both FA synthesis and FA oxidation are stimulated at the same time [94,95], so this may be the case also in HCC. Another study showed increased percent of MUFAs and decreased PUFAs levels in HCC tumors compared to normal liver tissue [91]. In a shotgun lipidomic analysis, Lin et al. [96] identified more than 1700 lipid compounds in HCC cell lines with various metastatic potential, including a non-metastatic hepatoma cell line (Hep3B), lowly metastatic cells (97L), and highly metastatic cells (LM3). This study showed that 93 significantly changed lipid species and decreased palmitic acyl group-containing glycerophospholipids were positively associated with the ability for metastasis of HCC cells. They also found that supplementation of palmitic acid inhibited the growth of HCC cells in contrast to normal hepatic cells, as well as distinctly reduced cancer cell invasiveness and migration [96]. However, Jion Ling [97] suggested that this should be verified with lower concentrations of palmitate, since the concentrations used in this study caused apoptosis, and it is hard to assess cell invasiveness and migration in such conditions.

On the other hand, HCC patients have elevated levels of acylcarnitines in their blood compared with healthy subjects [83,90]. This suggests impaired FA oxidation and a deficiency of carnitine palmitoyltransferase 2 (CPT2), which metabolizes acylcarnitines to acyl-CoA in mitochondria. It was also postulated that the blood concentrations of acylcarnitines may be used as a potential biomarker of HCC [83]. Short- and medium-chain acylcarnitines and free carnitines increased with the progression of malignant liver diseases [90].

The above-discussed HCC-related lipid alterations are summarized in Table 1.

Lipidomics based on mass spectrometry is usually an applied technique to record alterations of the lipid profile in many cancer/tumor matrices. Therefore, lipidomic studies have a potential to improve prevention, early detection, and targeted therapy of HCC.

## 7. Conclusions

NAFLD is associated with lipid alterations both on the level of liver and serum lipid composition as well as on the level of the expression of genes related to lipid metabolism in hepatocytes. A thorough understanding of these disorders creates an opportunity to establish new diagnostic methods and therapeutic goals based on lipid metabolism in NAFLD patients. Furthermore, HCC, a frequent late complication in NAFLD patients, is associated with alterations of lipid metabolism, and the above conclusions also apply to this condition.

## Figures and Tables

**Table 1:** Lipid alterations in hepatocellular carcinoma (HCC).

Lipid Molecule	Used Matrix	Patients/ Experimental Model	Direction of Change	Reference
16:0 MUFA 16:1	FAs in plasma phospholipids	HCC patients	↓	[91]
	tumor tissue	HCC patients	↑	[91]
	FFAs in plasma	HCC patients	↑	[90]
18:1 n-9 PUFA	FAs in plasma phospholipids	HCC patients	↓	[91]
	tumor tissue	HCC patients	↓	[91]
18:2 n-6 18:2 n-6 18:3 n-3 20:4 n-6	FAs in plasma phospholipids	HCC patients	↓	[91]
	FFAs in plasma	mouse model	↓	[92]
	FFAs in plasma	mouse model	↓	[92]
	FAs in plasma phospholipids	HCC patients	↓	[91]
20:5 n-3 22:6 n-3	FFAs in plasma	mouse model	↓	[92]
	FFAs in plasma	mouse model	↓	[92]
acylcarnitines	serum samples	HCC patients	↑	[83,90]
phosphatidylcholines (PCs)	serum samples	HCC patients	↓	[93]
phosphatidylserines (PSs)	serum samples	HCC patients	↓	[93]
phosphatidylinositols (PIs)	serum samples	HCC patients	↓	[93]
